# Bioremediation of Landfill Leachate with Fungi: Autochthonous vs. Allochthonous Strains

**DOI:** 10.3390/life8030027

**Published:** 2018-07-04

**Authors:** Federica Spina, Valeria Tigini, Alice Romagnolo, Giovanna Cristina Varese

**Affiliations:** Department of Life Sciences and Systems Biology, University of Turin, viale Mattioli, 25, 10125 Turin, Italy; federica.spina@unito.it (F.S.); valeria.tigini@unito.it (V.T.); alice.romagnolo@unito.it (A.R.)

**Keywords:** biodegradation, autochthonous fungi, leachate, detoxification

## Abstract

Autochthonous fungi from contaminated wastewater are potential successful agents bioremediation thanks to their adaptation to pollutant toxicity and to competition with other microorganisms present in wastewater treatment plant. Biological treatment by means of selected fungal strains could be a potential tool to integrate the leachate depuration process, thanks to their fungal extracellular enzymes with non-selective catalytical activity. In the present work, the treatability of two real samples (a crude landfill leachate and the effluent coming from a traditional wastewater treatment plant) was investigated in decolorization experiments with fungal biomasses. Five autochthonous fungi, *Penicillium brevicompactum* MUT 793, *Pseudallescheria boydii* MUT 721, *P. boydii* MUT 1269, *Phanerochaete sanguinea* MUT 1284, and *Flammulina velutipes* MUT 1275, were selected in a previous miniaturized decolorization screening. Their effectiveness in terms of decolorization, enzymatic activity (laccases and peroxidases), biomass growth and ecotoxicity removal was compared with that of five allochthonous fungal strains, *Pleurotus ostreatus* MUT 2976, *Porostereum spadiceum* MUT 1585, *Trametes* *pubescens* MUT 2400, *Bjerkandera* *adusta* MUT 3060 and *B. adusta* MUT 2295, selected for their well known capability to degrade recalcitrant pollutants. Moreover, the effect of biomass immobilization on polyurethane foam (PUF) cube was assessed. The best decolorization (60%) was achieved by *P. spadiceum* MUT 1585, *P. boydii* MUT 721 and MUT 1269. In the first case, the DP was achieved gradually, suggesting a biodegradation process with the involvement of peroxidases. On the contrary, the two autochthonous fungi seem to bioremediate the effluent mainly by biosorption, with the abatement of the toxicity (up to 100%). The biomass immobilization enhanced enzymatic activity, but not the DP. Moreover, it limited the biomass growth for the fast growing fungi, MUT 721 and MUT 1269. In conclusion, robust and versatile strains coming from well-characterized collections of microorganisms can obtain excellent results comparing and even exceeding the bioremediation yields of strains already adapted to pollutants.

## 1. Introduction

During the past several decades, risks associated with the pollution caused by landfill leachate became more and more evident, for both the environment and the human health [1,2,3,4]. Landfill leachates are characterized by harsh conditions i.e., high concentration of ammonia and recalcitrant xenobiotics and low BOD/COD ratio, which are at the base of the failure of their treatment in conventional plants [5,6,7,8,9,10]. The ineffectiveness of treatments results in the persistence of the dark color and toxicity in effluents coming out from wastewater treatment plants [5,11].

Besides the concern about chemical pollution, a greater consciousness has been created regarding the sanitary risk due to the presence of dangerous microorganisms [12]. This also includes several fungi, which are considered emerging pathogens [3]. Recently, the presence of uncultured parasites belonging to the phylum Cryptomycota was reported in concentrated medium aged landfill leachate, which has high NH+-N content [13]. On the other hand, autochthonous microorganisms could be a precious resource for the bioremediation of the leachate itself. Autochthonous microorganisms, indeed, are necessarily tolerant against the high toxicity of the leachate and may also take part in the degradation of the recalcitrant molecules [14,15].

Bioremediation operated with selected fungal strains can present several opportunities, overcoming some disadvantages of other treatment methods [16]. In particular, extracellular oxidative enzymes extruded by fungi have non-selective catalytic activity and take part in the degradation of recalcitrant compounds requiring a high redox potential [17]. Nevertheless, the wastewater toxicity and the maintenance of non-sterile conditions for long-term operation could limit even the most powerful biodegrading fungal strain [18].

Therefore, a successful exploitation of fungi in wastewater bioremediation is promoted by picking strong and adaptable strains. In particular, it can be dealt with by two strategies. First, the biodegradation capability of autochthonous fungal strains could be explored, since they are already adapted to this toxic environment and to competition with autochthonous bacteria [3,14,19]. Otherwise, allochthonous fungal strains coming from well-characterized collections of microorganisms could be used.

In the present work, two real samples (a crude landfill leachate and its effluent coming from a traditional wastewater treatment plant) were treated in decolorization experiments with a total of 10 fungal strains: five autochthonous fungi selected in a previous miniaturized decolorization screening [3,14] and five allochthonous fungal strains, selected for their capability to degrade and detoxify textile wastewater [20]. The effectiveness of the treatment was evaluated in terms of decolorization, COD, and toxicity removal.

## 2. Materials and Methods

### 2.1. Samples

The two types of wastewater were sampled in a treatment plant located in Italy. The first (hereafter called “leachate”) consisted of a landfill leachate sampled before traditional treatment (nitro-denitro and biological oxidation by means of activated sludge). The second (hereafter called “effluent”) was a mix consisting of leachate (70% *v*/*v*) and other wastewater (30% *v*/*v*), and was sampled after biological oxidation and nitrification–denitrification treatment. Aliquots of both the leachate and the effluent were daily sampled for a period of 15 days in order to obtain more representative composite samples. Their chemical features were already described [3]. The chemical parameters exceeding the Italian legal threshold values are reported in Table 1.

### 2.2. Autochthonous Biomasses

Five fungi isolated from the samples were previously selected among 51 autochthonous strains as the most effective in color removal from the leachate and the effluent in a miniaturised screening [3]. They are preserved at the *Mycotheca Universitatis Taurinensis* (MUT, University of Turin, Department of Life Sciences and Systems Biology, Italy) with the following accession numbers: *Penicillium brevicompactum* MUT 793, *Pseudallescheria boydii* MUT 721, *P. boydii* MUT 1269, *Phanerochaete sanguinea* MUT 1284, and *Flammulina velutipes* MUT 1275.

### 2.3. Allochthonous Biomasses

*Pleurotus ostreatus* MUT 2976, *Porostereum spadiceum* MUT 1585, *Trametes pubescens* MUT 2400, *Bjerkandera adusta* MUT 3060 and *B. adusta* MUT 2295 were selected for their capability to degrade xenobiotics in simulated and real textile effluents [20].

### 2.4. Biodegradation Screening with Free Biomasses

The fungi were pre-cultured in Petri dishes contained Malt Extract Agar medium (MEA), containing 20 g/L glucose, 20 g/L malt extract, 20 g/L agar, 2 g/L peptone. After 4–7 days incubation, portions of mycelium were taken from the margins of the colony and homogenized in liquid culture media in the ratio of 1 cm^2^ per mL, according to the kind of fungus: a high nitrogen content medium, GHY (10 g/L glucose and 3.8 g/L yeast extract) for basidiomycota and liquid Malt Extract medium (20 g/L glucose, 20 g/L malt extract, 2 g/L peptone) for ascomycota. The inoculum consisted in mycelial suspension aliquots (500 µL) put in 40 mL liquid cultural medium and incubated at 25 °C and 110 rpm [21].

After a week, the culture broth was replaced with 40 mL of untreated wastewater and the biomasses were incubated for an additional 7 days in the same environmental conditions. Controls consisting of wastewater without fungal biomass were also set up. All the trials were performed in three replicates. Color and enzymes were daily analyzed. COD and toxicity were estimated at the end of the experiment only.

### 2.5. Biodegradation Experimental with Immobilized Biomasses

The best biomasses selected in the previous experiment were tested in scaled free vs. immobilized biomasses tests. After a pre-culture phase in solid medium, as described before, all the biomasses were homogenized in liquid MEA (1 cm^2^/mL). This mycelium suspension (5 mL) was used as inoculum in 250 mL flasks containing 170 mL MEA. Eight replicates were performed for each fungus. Ten cubes (2 cm^3^) of polyurethane foam (PUF) were put in four out eight replicates. Moreover, an aliquot of 5 mL of each mycelium suspension was put in a dry oven for 24 h, in order to estimate the dry weight of the inocula.

After 7 days of incubation at 25 °C and 120 rpm, the culture broth was replaced with 100 mL of effluent and the cultures were incubated with the previous environmental conditions. Controls consisting of wastewater without fungal biomass were also set up. All of the trials were performed in three replicates. Color and enzymes were monitored daily. COD and toxicity were estimated at the end of the experiment only.

### 2.6. Enzymatic Activity Assay

The enzymatic activity was monitored with a spectrophotometer (TECAN Infinite M200), thanks to a colorimetric reaction, which reveals the oxidation mediated by the enzymes. For laccases, the oxidation of 2,2′-azinobis(3-ethylbenzothiazoline-6-sulfonic acid)(ABTS) was monitored at 420 nm in 0.1 M sodium citrate buffer (pH 3) at 25 °C [22]. For manganese-independent (MiP) and manganese-dependent (MnP) peroxidases, the oxidation of 3-dimethylaminobenzoic acid/3-methyl-2-benzothiazolinone hydrazone hydrochloride (DMAB/MBTH) was monitored at 590 nm in 0.1 M succinate lactate buffer (pH 4.5) at 25 °C [23]. MnSO4 (25 µM) was added to detect MnP. The results were converted in International units (U).

### 2.7. Ecotoxicity Tests

Ecotoxicity tests were performed before and after fungal treatments. Two bioassays were used, according to their high sensitivity [3].

The algae tests were performed according to the standard UNI EN ISO 8692: 2005 using a monospecies culture of *Raphidocelis subcapitata* (Korshikov) Nygoard, Komárek, Kristiansen and Skulberg. A cell suspension (2.5 × 10^4^ cell) was inoculated in 2.5 mL of different effluent dilutions (100%, 50%, 25%, 12.5%, 6.5%, 3.2%, 1.6% and 8%). The trial was performed in triplicate. Moreover, an abiotic control (effluent without the algal inoculum) and 6-replicated biotic control (algal inoculum in water without effluent) were performed.

After 48 h incubation at 23 °C under white light (8000 lux), the cell’s concentration was spectrophotometrically evaluated. A linear correspondence between the absorbance and the cell concentration, previously assessed, allowed the conversion. The inhibition of the algal growth was expressed as a percentage with respect to the average of the algal growth observed in the 6 biotic controls.

The dicotyledonous plant, *Lepidium sativum* L., was used for phytotoxicity tests, according to the standard method UNICHIM 1651: 2003. The seeds (90% germination warranty) were put in Petri dishes (9 cm diameter) containing 5 mL undiluted sample and a Whatman filter No. 1. The trials and the control (distilled water) were performed in 4 replicates.

The incubation was carried out for 72 h in the dark at 25 °C. Subsequently, the toxicity was expressed as germination index (IG) inhibition percentage compared to the IG average in controls.

### 2.8. Statistical Analyses

The nonparametric Mann–Whitney test was run to assess the significance (*p* ≤ 0.05) of the differences between both DP and toxicity values.

## 3. Results and Discussion

### 3.1. Biodegradation Screening with Free Biomasses

Despite the modification of the samples, the crude landfill leachate was too toxic to allow the growth and the metabolic activity of all fungi. All of the monitored parameters showed a substantial inactivity of fungi (data not shown). On the contrary, the treatment with fungal biomasses was effective against the effluent.

**Figure 1:**
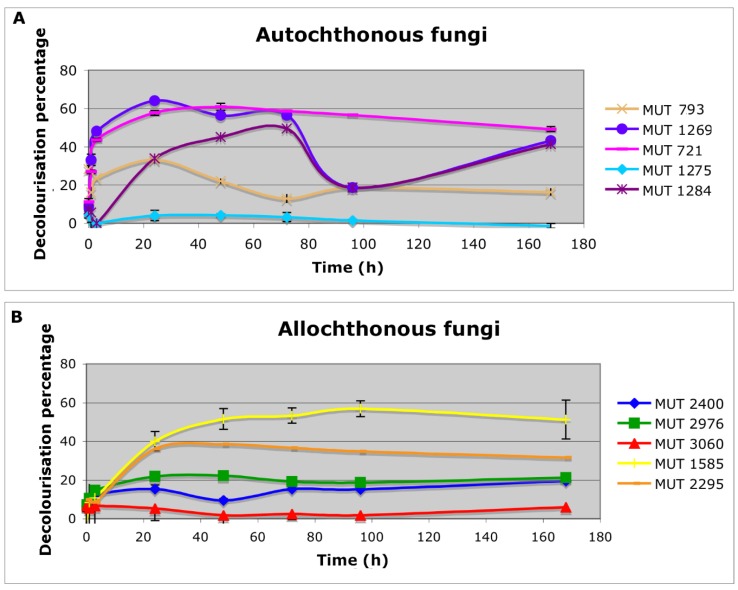
Effluent decolorization percentage achieved with both autochthonous (**A**) and allochthonous (**B**) fungal biomasses.

The autochthonous biomasses determined a rapid (within 6 h) decolorization of the effluent, ranging from 20% to 50% for *P*. *brevicompactum* MUT 793 and *P. boydii* MUT 1269, respectively. A sole exception is represented by *F. velutipes* MUT 1275 that did not cause any substantial color removal (Figure 1). These data are in contrast with literature evidence, which reported that this species could degrade tannins in coffee husk or coffee spent-ground [24]. Consequently, a decolorization of the leachate would have been expected, since it generally contains tannins [25].

Another potentially interesting genus, among the autochthonous species, is *Penicillium*. This genus has been showed to degrade different xenobiotics with low co-substrate addition, and are potentially interesting for feasible applications in wastewater bioremediation [26].

*P. brevicompactum* has been signalled to metabolize terbuthylazine, a toxic herbicide chemically similar to atrazine, considered one of the most persistent triazine herbicide in surface environments. As in the present study, the tested strain, which was isolated from a pesticide-primed soil, has presumably acquired this biodegradation capability through chronic exposure to the pollutant [27]. The exposition to pollutants results in the selection of microorganisms with high bioremediation potentiality even in the coastal waters [28]. The marine ecosystem can share different fungal species with landfill leachate (e.g., *Arthrinium sphaerospermum*, *Cladosporium cladosporioides*, *Candida parapsilosis, Rhodotorula* spp.), and several species can be human pathogens [3,28]. Both of these environments are characterized by wide variations in the salt concentration, on account of evaporation or rainfall. A relatively high salt concentration is an environmental condition that supports the colonization of opportunistic pathogens [29]. Thus, marine habitats and human impacted water, e.g., wastewater, can represent a selective substrate for the development of pathogens [30].

*P. boydii* complex species is a typical example of opportunistic fungal pathogens that are common in human impacted areas, and displays high biodegradation potential [31,32]. Among the autochthonous strains, *P. boydii* MUT 1269 achieved the highest and fastest DP (Figure 1). However, the instability of the color removal (DP decreased at 20% after three days) suggested the involvement of the biosorption process rather than biodegradation.

Allochthonous biomasses determined a slower decolorization, achieving a pick within 1–4 days. *P*. *spadicea* MUT 1585 achieved the highest color removal (51% within seven days). *P. ostreatus* MUT 2976 and *T. pubescens* MUT 2400 showed comparable decolorization yields (DP around 20%). The two strains belonging to the species *B. adusta*, MUT 3060 and MUT 2295 showed significantly different decolorization capacity (4% and 40%, respectively). The great diversity in decolorization yields indicates that the degradation capabilities are strain-specific rather than species-specific. The different behavior of these two strains was already observed towards textile wastewater [33].

It is worth noting that allochthonous basidiomycota showed physiological versatility that allows them to remain active in these extreme environmental conditions and to compete with the autochthonous microorganisms in the effluent. Actually, from this effluent, a variety of filamentous fungi, bacteria and algae was found [14]. The fungal competitiveness is an essential feature for the effectiveness of bioremediation treatment applied to real wastewater, in which autochthonous microorganisms are already acclimated to the environmental condition. The competition with bacteria is indeed one of the main causes of failure of fungal application in wastewater treatment plants. When tested on real wastewater, the selected fungal strain is often not able to compete with autochthonous microorganisms [34]. Bacteria may compete with fungi for carbon and nutrient sources thanks to their faster growth rate and quickly colonize reactors. Their contamination is associated with fungal growth suppression, mycelium disruption and a drop in enzymatic production, which affect pollutant removal performance [35,36]. In addition, in non-sterile conditions, an increase in the toxicity may also occur, as the microbial consortium developed in a bioreactor can have alternative degradation pathways and metabolites. Therefore, finding reactor configurations for minimizing adverse effects of microbial colonization is a basilar need [34]. In this regard, the addition of selective co-substrate, characterized by recalcitrance to bacterial degradation and high bacterial toxicity, could be useful to obtain a successful fungal colonization even in scaled-up non-sterile conditions [37].

**Figure 2:**
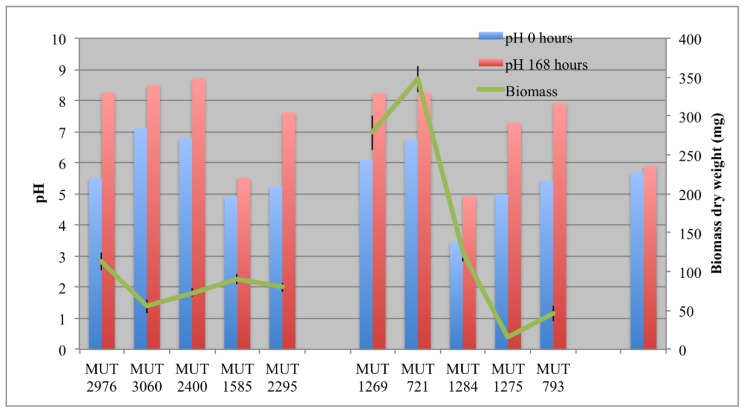
Biomass developed at the end of the experiment, and pH at the beginning (0 h) and at the end (168 h) of the experiment.

The biomass developed in each bioreactor is reported in Figure 2. Among the autochthonous strains, *P. boydii* MUT 1269 and MUT 721 were the fastest growing strains, with 0.35 g dry weight developed within seven days. Since the biosorption process was hypothesized for the DP caused by these two isolates, the rapid biomass development probably enhanced the decolorization effectiveness. On the contrary, *F. velutipes* MUT 1275 showed the lowest growth rate. Possibly, the wastewater may inhibit in some way the growth of this strain. However, the fungal strain showed a low growth rate even in the pre-culture phase in solid medium (data not shown). The low growth rate could be ascribable to different phenomena, closely related to the need of a different optimal environment to trigger *F. velutipes* growth. According to the literature, this species is known as winter mushroom, requiring cold conditions (−2 to 14 °C) to develop the fruit-body and maximal mycelium growth at 25 °C [38]. Nevertheless, the low rate could be due, more likely, to the culture medium. *F. velutipes* growing better in the presence of potatoes’ dextrose than malt extract [39].

During the experiment, a gradual increase of the pH, even over 8, was observed in the bioreactors (Figure 2). Only for *P*. *spadicea* MUT 1585 was the increase not significant. In abiotic control, the pH remained stable.

**Figure 3:**
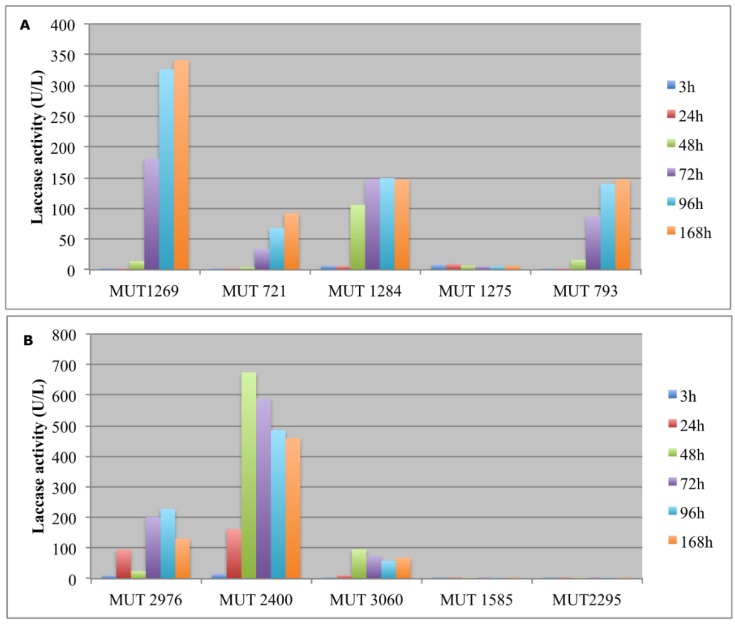
Laccases activity (U/L) detected in the extracellular supernatants of autochthonous (**A**) and allochthonous (**B**) fungi during the experiment.

**Figure 4:**
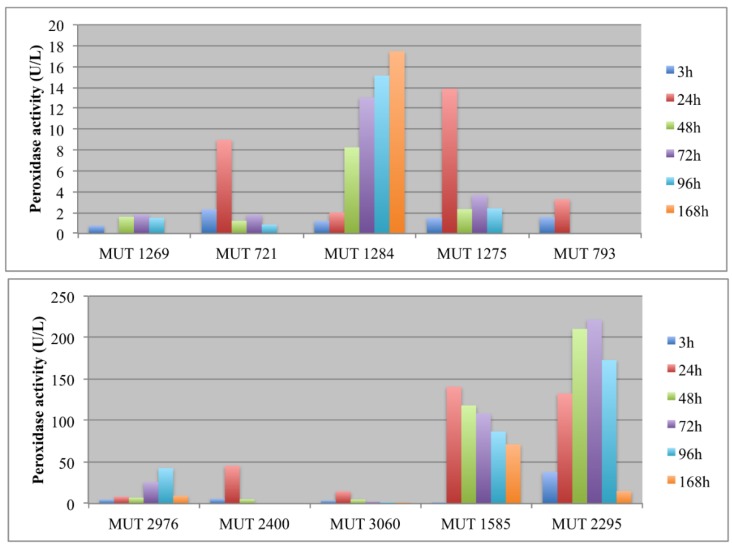
Peroxidase activity (U/L) detected in the extracellular supernatants of autochthonous (**A**) and allochthonous (**B**) fungi during the experiment.

The enzymatic activity was monitored in terms of laccases and peroxidases (Figure 3 and Figure 4). The production of large amount of laccases by autochthonous strains occurred from the fourth day. In order to evaluate the catalytic effectiveness of the produced enzymes during the experiment, it must be kept in mind that these enzymes are mainly active at acidic pH. As a consequence, it is possible that laccases were weakly active since the pH rapidly increased up to 8.

As regards the allochthonous strains, *T. pubescens* MUT 2400 showed a typical inductive mechanism, producing a huge amount of laccases after 24–48 h. It is well known from the literature, indeed, that the production of some kinds of oxidative enzymes by basidiomycota is due to a secondary metabolism activated by the presence of aromatic compounds [40].

A high peroxidase activity was produced only by *P*. *spadicea* MUT 1585 and *B. adusta* MUT 2295. As regards this latter strain, the peroxidase activity was very variable, showing a severe decrease of enzyme production at the end of the experiment (Figure 4a). Peroxidases are oxidative enzymes with an optimum of activity in acidic environments (around pH 4.5–5.5) and could be induced by phenolics in wastewater [41]. Thus, the high production of peroxidases by *P*. *spadicea* MUT 1585, coupled to the strain capacity of maintaining the wastewater pH near a neutral value, is noteworthy. Peroxidases from white rot fungi, contrarily to laccase, demonstrated to be stable in the treatment of high phenolic content wastewater such as olive mill wastewater [41].

In the recent literature, different basidiomycota (e.g., *Trametes versicolor*, *Dichomitus squalens*, *Trametes menziesii*, *Ganoderma australe*) have been investigated for landfill decolorization and detoxification with the involvement of oxidative enzymes [10,42,43,44]. The results are not always comparable since, sometimes, different parameters were monitored (COD and BOD instead of color and toxicity, phenolic content). However, when decolorization was monitored, it was around 60–78%. Autochthonous basidiomycota were already pointed out as interesting bioremediation agents, thanks to their oxidative enzymes. *P. sanguinea* was indicated as a promising biotechnological agent, being capable of performing a highly selective degradation of lignin [45,46]. *F. velutipes* has been demonstrated to produce ligninolitic enzymes, but with higher yields before fruiting [47]. This could explain the low enzymatic activity recorded for this species in the present work. It is worth noting that the allochthonous basidiomycetes were significantly more effective both in laccase and in peroxidase production, achieving a landfill leachate DP comparable to the yields in the literature [48]. This indicates that a long-lasting and detailed selection of fungal strains with high enzyme expression is of extreme importance for biotechnological application. This allows for identifying, indeed, fungi with a great enzymatic and physiological versatility, exceeding bioremediation potential of well acclimated organisms.

However, even ascomycetes have been reported to degrade recalcitrant compounds thanks to oxidative enzymes. In particular, *Penicillium* produced laccases and peroxidases [49]. In this study, the most effective autochtonous fungus in laccase production was a *P. boydii* strain. It is worth noting that, in a few reports, laccase production has been associated with *P. boydii* species complex. One previous paper studied the ligninolytic enzymes’ production of *Pseudallescheria angusta*, highlighting the presence of laccase, LiP and MnP [50]. In another work, the laccase production by *Scedosporium apiospermum* was studied in relation to the PCBs removal, but its role in the degradation of these pollutants is still unclear [51].

As regards the ecotoxicological assessment, the two model organisms responded both to the toxic compounds in the effluent and, as expected, also to the pH. The change in the pH value (from pH 8.5 to 5) by means of acid addition triggered a decrease of the sample toxicity towards both bioassays. According to the algal test, the toxicity decreased of 30% (considering a 12% of sample dose); however, according to the phytotoxicity test, the toxicity decreased of 40% (considering the 100% sample dose).

The results about ecotoxicity variation due to the fungal treatment are reported in Table 2. They were elaborated with respect to the sample with unmodified alkaline pH.

At least for one test, the toxicity was reduced, up to a complete removal of the toxicity (MUT 721). However, it was difficult to clearly correlate the toxicity to the monitored parameters (e.g., DP or enzymes).

Changes in toxicity after fungal treatment of landfill leachate have been rarely evaluated [42,52]. Generally, the toxicity decreased after the fungal treatment. In addition to the decrease of pollutants, changes in pH and electrical conductivity also had a role in the detoxification. In the present case, a close correlation between chemical parameters (decolorization, enzymatic activity, pH) and toxicity variation was absent.

Interestingly, the treatment with the two *P. boydii* strains resulted in significantly different toxicity variation recorded by *R. subcapitata*. Since the final DPs obtained by the two strains were comparable (Figure 1), probably colored pollutants (e.g., humic acids, tannins, etc.) were similarly removed by these strains. If so, the difference in toxicity could be ascribable to the different produced secondary metabolites due to the different expressed enzymatic pathway (e.g., laccases) (Figure 3). Laccase, as other substances that catalyze an oxidation reaction [53], could expose the alga to oxidative stress and cellular damage inducing a growth rate decrease.

### 3.2. Biodegradation Experiment with Immobilized Biomasses

**Figure 5:**
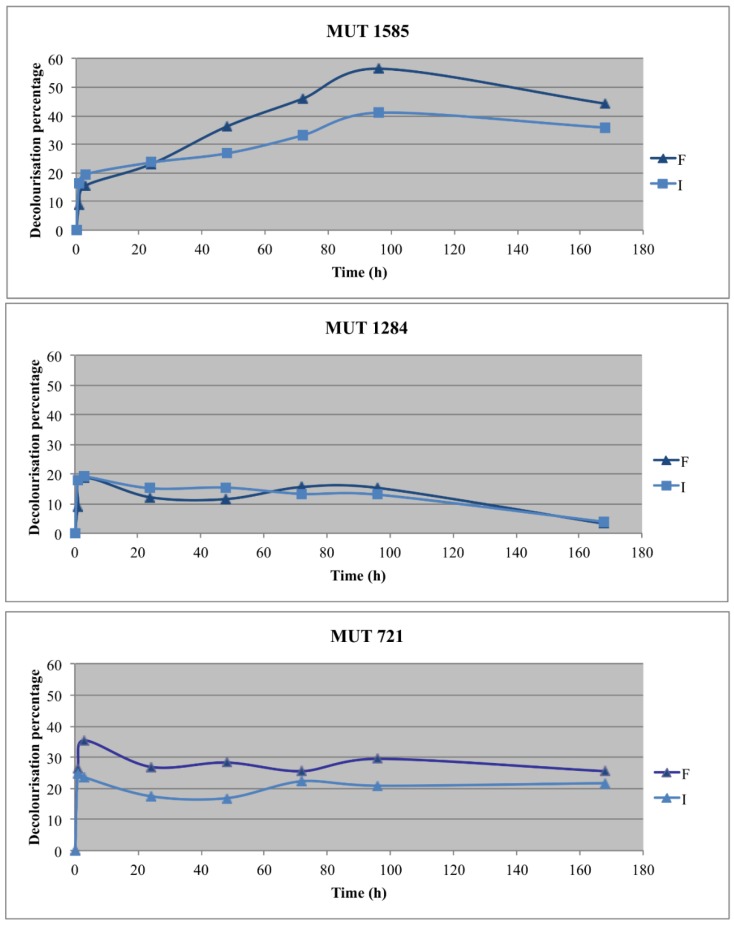
Effluent decolorization percentage achieved with the three selected strains in the two experimental lines (F = free; I = immobilized).

Two autochthonous fungi, *P. sanguinea* MUT 1284 and *P. boydii* MUT 721, and one allochthonous fungus, *P. spadiceum* MUT 1585, were selected for the optimization experiments with the fungal biomass immobilization. The effluent decolorization was recorded in all the experimental lines (Figure 5).

The best decolorization rate was obtained with the free biomass of *P. spadiceum* MUT 1585, which achieved 50% decolorization at the end of the experiment. Its immobilized biomass showed a (DP up to 40%, significantly lower than the free biomass. *P. sanguinea* MUT 1284 and *P. boydii* MUT 721 achieved a decolorization rate between 28% and 35%, within the first 3 h, without any significant difference between immobilized and free biomasses. After this period, the decolorization percentage decreased up to 3% for *P. sanguinea* MUT 1284, whereas, for *P. boydii* MUT 721, the decolorization percentage remained unchanged. The results indicate that the decolorization is probably due to a main biosorption process and a metabolic transformation of chromophore molecules could be a further step of the fungal treatment. Actually, biosorption is a fast, passive and reversible process [54]. This hypothesis is confirmed by the aspect of the biomasses, which was deeply colored at the end of the experiment. On the contrary, the biomass of *P. spadiceum* MUT 1585 was uncolored (data not shown).

The immobilization process affected the biomass development and the biomass recover at the end of the experiment. The immobilized biomass developed a higher biomass weight (2.3–2.6 g dry weight) with respect to the free biomass (0.5–0.8 g dry weight). From an applicative point of view, the biomass immobilization displays many advantages, increasing enzyme production, allowing several continuous cycles, avoiding clogging problems caused by a free biomass and reducing time and resources spent for the separation of the mycelium, by means, for example, of ultra filtration membranes [55]. This is a very important aspect in real application, since clogging issues due to the presence of floating free biomass affecting some reactor configurations.

The pH of abiotic control remained unchanged. A gradual increase of the pH values was recorded during the experiment, up to more than seven for immobilized autochthonous biomasses. On the contrary, *P. spadiceum* MUT 1585 maintained the pH around 5 in both the cultural condition, demonstrating the ability of this strain to remain metabolically active in the harsh condition of the effluent.

**Figure 6:**
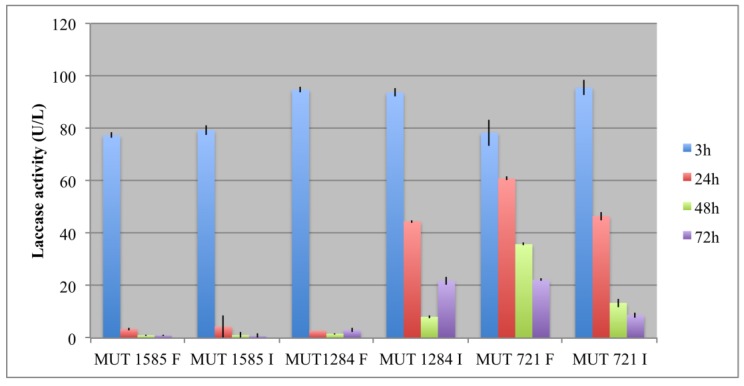
Laccase activity (U/L) expressed by free and immobilised fungal biomasses during the experiment.

All of the fungal strains showed an interesting laccase activity, with higher values within the first 3 h both as free and immobilized biomass (Figure 6). In particular, *P. sanguinea* MUT 1284 and *P. spadiceum* MUT 1585 showed no significant difference between free and immobilized biomasses. On the contrary, *P. boydii* MUT 721 expressed a higher laccase activity as immobilized biomass. This strain showed a more gradual decrease of laccases activity in the later hours with respect to the other strains. However, the decrease was more rapid in the immobilized form.

Peroxidase activity was monitored only for *P. spadiceum* MUT 1585 on account of literature data and previous results, which indicated only this strain as a peroxidase producer. The cultural condition affected the enzymatic production; actually, free biomass showed higher peroxidase production (up to three times). It is worth noting that peroxidase activity increased after the first 3 h and achieved the maximum at the end of the experiment in both of the cultural conditions (up to 140 U/mL and 70 U/mL for free and immobilized biomasses, respectively).

Thus, probably laccase was produced in the pre-growth phase and then it was inhibited or not stimulated in the treatment phase. On the contrary, peroxidases seem to be induced by leachate in *P. spadiceum* MUT 1585. Moreover, they have a higher redox potential with respect to laccase that could results in a greater biodegradation capability.

*R. subcapitata* test recorded an increase of toxicity in both the cultural conditions. The toxicity variation was elaborated on the base of the results at 6.25% dilution (Table 3).

As regards the ecotoxicity with *L. sativum*, an increase of toxicity in both of the cultural conditions was recorded. The toxicity variation was elaborated on the base of the results at 6.25% dilution (Table 3). An *R. subcapitata* test was more sensitive to the effluent composition, with responses strongly dependent on the occurred treatment. The autochthonous strains *P. sanguinea* MUT 1284 and *P. boydii* MUT 721 caused a decrease in the effluent toxicity in free and immobilized conditions, respectively. This could be due to the biosorption process that occurred in the treatment with these strains. On the contrary, *P. spadiceum* MUT 1585 increased the effluent toxicity in both of the culture conditions. In this case, the biotransformation of the pollutants may have produced more toxic compounds that could be responsible for the toxicity increase (Table 3).

The COD was measured only for the cultural lines that determined a substantial decolorization of the effluent (free and immobilized biomasses of *P. spadiceum* MUT 1585). At the beginning of the experiment, the COD values were 2530 mg/L and slightly decreased after the treatment with the immobilized biomass (2320 mg/L), whereas they remained substantially unmodified with the free biomass (2550 mg/L). These results seem to indicate that decolorization does not necessarily imply the degradation of pollutant and probably a more longer treatment could be useful to achieve a decrease in COD.

## 4. Conclusions

In the present work, the treatability of two real samples (a crude landfill leachate and the effluent coming from a traditional wastewater treatment plant) was investigated. The crude leachate did not allow active fungal growth. The treatment of the effluent with autochthonous fungi *Pseudallescheria boydii* MUT 721, *Phanerochaete sanguinea* MUT 1284, achieved good and very fast decolorization percentage (60%), coupled by detoxification. Biosorption seems to be the main process involved. Their decolorization effectiveness was comparable to that of allochthonous *Porostereum spadiceum* MUT 1585. In this last case, nevertheless, the decolorization was slower and caused the increase of toxicity. It was likely that the biodegradation process occurred here, with the involvement of laccases and peroxidases. Moreover, the effect of biomass immobilization on polyurethane foam (PUF) cube was assessed. The biomass immobilization is needed in order for a scale up of the process, since it generally enhances enzymatic activity, and limits the biomass growth. In conclusion, autochthonous fungal strains are valid candidates for bioremediation of landfill leachate. However, the mechanism involved should be more deeply investigated, in order to optimize bioremediation yields. Moreover, the impact of the employment of opportunistic pathogens should be considered in view of a real application in a wastewater treatment plant. On the other hand, robust and versatile strains coming from well-characterized collections of microorganisms can obtain excellent results comparing and even exceeding the bioremediation yields of strains already adapted to pollutants. In the case of versatile and robust allochthonous fungal strains, landfill leachates are selective substrates that could favor the development of fungal biomasses in real and non-sterile conditions.

## Figures and Tables

**Table 1:** Sample chemical parameters exceeding the Italian legal threshold.

Parameter	Leachate	Effluent	Legal Threshold Limit
Cuprum (as Cu)	0.60 mg L^−1^	0.15 mg L^−1^	0.1 mg L^−1^
Zinc (as Zn)	0.66 mg L^−1^	1.38 mg L^−1^	0.5 mg L^−1^
Total hydrocarbons	<100 mg L^−1^	598.7 mg L^−1^	5 mg L^−1^
Ammonium (as NH_4_)	2266.0 mg L^−1^	408.0 mg L^−1^	15 mg L^−1^
Total nitrogen (as N)	2537.9 mg L^−1^	514.1 mg L^−1^	20 mg L^−1^
Chlorides (as Cl^−^)	3193 mg L^−1^	2550 mg L^−1^	1200 mg L^−1^
Anionic surfactants (MBAS)	23.07 mg L^−1^	37.9 mg L^−1^	2 mg L^−1^
Non ionic surfactants (PPAS)	23.48 mg L^−1^	17.2 mg L^−1^	
Cationic surfactants	<10 mg L^−1^	<10 mg L^−1^	
COD (as O_2_)	6166 mg L^−1^	2099 mg L^−1^	160 mg L^−1^
BOD_5_ (as O_2_)	4209 mg L^−1^	1410 mg L^−1^	40 mg L^−1^
Suspended solids	1064 mg L^−1^	604 mg L^−1^	80 mg L^−1^
Colour	visible at 1:20 dilution	visible at 1:20 dilution	not visible at 1:20 dilution

Since the unmodified samples proved to not be able to support fungal growth as such (data not shown), their pH was adjusted from around 8 to 5, and 0.1 g/L glucose was added. The decolorization percentage (DP) was estimated spectrophotometrically (TECAN Infinite M200) as the decrease of the functional integration of spectra in the range of 360–790 nm.

**Table 2:** Variation of toxicity for *R. subcapitata* and *L. sativum*, after the fungal treatment with respect of the untreated effluent.

		*R. subcapitata*	*L. sativum*
Autochthonous fungi	MUT 1269	−7%	−39%
	MUT 721	−100%	−40%
	MUT 793	−53%	−4%
	MUT 1275	69%	−49%
	MUT 1284	211%	−13%
Allochthonous fungi	MUT 2400	211%	−5%
	MUT 1585	42%	−35%
	MUT 2295	−72%	−44%
	MUT 2976	−48%	−35%
	MUT 3060	−33%	−16%

**Table 3:** Variation of the effluent ecotoxicity (at the doses 100% and 6.25 for *L. sativum* and *R. subcapitata,* respectively) after the treatment with free and immobilised biomasses.

Fungal Cultures	*L. sativum* 100%	*R. subcapitata* 6.25%
Free biomass MUT 1585	74.9%	65.7%
Free biomass MUT 1284	82.3%	−60.9%
Free biomass MUT 721	24.3%	−0.9%
Immobilised biomass MUT 1585	74.8%	29.1%
Immobilised biomass MUT 1284	11.0%	−2.9%
Immobilised biomass MUT 721	45.0%	−13.7%
